# Untargeted LC–MS Plasma Metabolomics Reveals Altered Amino Acid and Carbohydrate Metabolism in Dairy Calves Supplemented with Direct-Fed Microbials

**DOI:** 10.3390/metabo16070441

**Published:** 2026-06-25

**Authors:** Oludotun O. Adelusi, David P. Casper, John O. Adebayo, Ahmed E. Kholif, Ibukun M. Ogunade, Uchenna Y. Anele

**Affiliations:** 1 Department of Animal Sciences, North Carolina Agricultural and Technical State University, Greensboro, NC 27411, USA; ooadelusi@aggies.ncat.edu (O.O.A.); joadebayo@aggies.ncat.edu (J.O.A.); aekholif@ncat.edu (A.E.K.); 2 Casper’s Calf Ranch, Freeport, IL 61032, USA; dpcasper@ncat.edu; 3 Dairy Science Department, National Research Centre, 33 Bohouth St. Dokki, Giza 12622, Egypt; 4 Division of Animal and Nutritional Science, West Virginia University, Morgantown, WV 26505, USA; ibukun.ogunade@mail.wvu.edu

**Keywords:** dairy calves, metabolome, direct-fed microbials

## Abstract

**Highlights:**

**What are the main findings?**

- Direct-fed microbial supplementation (individual or combined *Lactobacillus plantarum*, *Bifidobacterium animalis*, and *Lactobacillus animalis*) significantly altered the plasma metabolome of pre-weaned dairy calves, with 24 metabolites differentially abundant compared with the control.
- Key metabolic pathways affected included branched-chain amino acid degradation (valine, leucine, isoleucine) and carbohydrate metabolism (pentose and glucuronate interconversions), indicating treatment-specific modulation of energy and nutrient metabolism.

**What are the implications of the main findings?**

- Direct-fed microbials can modulate metabolic pathways associated with energy balance and amino acid utilization, suggesting a mechanistic basis for improved resilience and metabolic efficiency in early-life calves.
- The identified metabolite biomarkers and enriched pathways provide potential targets for developing nutritional strategies to mitigate metabolic stress and support health during the pre-weaning period.

**Abstract:**

**Background/Objectives**: Direct-fed microbials (DFMs) are widely used in dairy calves to improve gut health and mitigate neonatal disorders, yet their systemic metabolic effects remain poorly defined. This study evaluated the impact of DFM supplementation on the plasma metabolome of pre-weaned dairy calves using untargeted liquid chromatography–mass spectrometry (LC–MS). **Methods**: Eighty-six Holstein bull calves (2 to 5 days old) were assigned to one of four treatments in a 2 × 2 factorial randomized complete block design: *Lactobacillus plantarum* in starter (CLP), a culture mix of *Bifidobacterium animalis* and *Lactobacillus animalis* in milk replacer (BBCM), and a combination of both (CMLP), or no supplementation (CON). Blood samples collected on days 0 and 56 were subjected to metabolomic profiling, and metabolites were annotated using Human Metabolome Database and Kyoto Encyclopedia of Genes and Genomes databases. **Results**: A total of 231 plasma metabolites were detected. Compared with CON, 24 metabolites were differentially abundant in DFM-treated calves (fold change ≥ 1.2 or ≤ 0.83; *p* ≤ 0.05). Supplemented calves exhibited increased abundances of ketone functional groups, aldehydes and amino acid-related metabolites. Metabolite set enrichment analysis identified 11 significantly enriched pathways. Branched-chain amino acid degradation pathways (valine, leucine, and isoleucine) were enriched in CLP and CMLP calves, whereas carbohydrate metabolism pathways, including pentose and glucuronate interconversions, were enriched in the CLP and BBCM groups. **Conclusions**: These findings demonstrate that DFM supplementation modulates systemic metabolism in dairy calves, particularly pathways involved in amino acid and carbohydrate utilization, suggesting enhanced metabolic efficiency during early life.

## 1. Introduction

Diarrhea is the most prevalent adverse health event affecting pre-weaned calves in North America, Europe, and Australia, responsible for 56.4% of pre-weaned heifer deaths and prompting 64.1% of operations to administer antimicrobials to treat diarrhea/digestive disease [1]. Even when treated with antimicrobials, early-life diarrhea and pneumonia impair long-term productivity and longevity of dairy calves [1,2,3]. Consequently, antibiotic alternatives that support health and improve growth during this stressful period need further evaluation. Direct-fed microbials (DFMs) are live microorganisms that confer health benefits when administered in adequate amounts. Strains such as *Lactobacillus plantarum*, *Bifidobacterium animalis*, and *Lactobacillus animalis* support stable gut microbiota, inhibit pathogen growth, and can improve calf growth [4,5]. Diarrhea causes dehydration, electrolyte imbalance, metabolic acidosis, and hypovolemia, which can lead to kidney failure, heart block, septicemia, and compensatory reduction in urine output [6]. It also induces intestinal microbial imbalance and gut dysbiosis, which can increase trimethylamine N-oxide, a metabolite of choline produced by gut microbiota and hepatic flavin monooxygenases, altering circulating metabolite profiles [6].

Metabolomics involves the comprehensive analysis of endogenous small molecule alterations to assess metabolic responses to stimuli and is a promising approach to understand metabolic regulation and alleviate disease-associated complications [7]. Blood plasma is widely used in bovine metabolomics due to its accessibility and its role as a functional readout of systemic metabolism [8]. The blood metabolome reflects ruminal microbial lipid metabolism, intestinal digestive and absorptive capacity, and fatty acid metabolism in the liver, adipose tissue, and muscle [9]. Metabolomics enables rapid detection of physiological or pathological changes, aiding identification of diagnostic markers and pathogenic mechanisms in diarrheic calves [8]. Principal component analysis shows significant metabolite differences between healthy and diarrheic calves. These biomarkers, linked to mineral absorption, protein digestion, and other pathways, may enable targeted interventions for the diagnosis and management of calf diarrhea [10]. Diarrheic calves exhibit decreased taurocholate and glucose and increased inflammation [11]. Elevated serum D-lactate, a bacterial fermentation byproduct, correlates with intestinal barrier dysfunction; damage to mucosa increases permeability, facilitating the release of D-lactate into bloodstream, where it accumulates due to limited hepatic metabolism [12,13]. DL-lactate decreased following *Pueraria* polysaccharide treatment, though its chiral structure was not distinguished [11].

We hypothesized that supplementation with a DFM program containing *Lactobacillus plantarum*, *Bifidobacterium animalis* and *L. animalis* would enhance the nutritional status and metabolic efficiency of calves by modulating systemic metabolism. Therefore, this study aimed to characterize the plasma metabolite profiles of calves receiving DFM supplementation, identify differentially abundant metabolites and determine the metabolic pathways associated with the observed metabolic response.

## 2. Materials and Methods

### 2.1. Study Ethical Approval

This metabolomic investigation was conducted as part of a broader calf feeding study designed to evaluate a DFM program, and the performance data are presented and discussed separately to allow for a more comprehensive effect of dietary interventions. The study was conducted at Casper’s Calf Ranch, LLC (Freeport, IL, USA), between 26 April 2023 and 20 June 2023, spanning a 56-day period. The management and feeding of the calves were carried out in accordance with the standards published in the fourth edition of Guide for the Care and Use of Agricultural Animals in Research and Teaching (ADSA-ASAS-PSA, 2020). To ensure licensed veterinary care and supervision, the operations of Casper’s Calf Ranch were carried out with the assistance of Lena Veterinary Clinic (Dr. Brandon and Morgan Scharping; Lena, IL, USA).

### 2.2. Animals, Experimental Design, and Diet Composition

The current experiment was conducted using facilities, procedures, treatment protocols, and sample collection methods similar to those employed in previously reported studies conducted at this contract research facility [5,14,15]. Eightysix Holstein bull calves (2 to 5 days old) sourced from various Wisconsin livestock auction barns via a commercial calf buyer (Knueppel Livestock, Shawano, WI, USA) were delivered in one lot on the night of 25 April 2023. Calves were assumed to have been fed colostrum at their respective dairy farm, but information on colostrum management and feeding was not available. Calves were vaccinated intranasally upon arrival with Nasalgen 3-PMH (Zoetis Inc., Parsippany, NJ, USA) and checked for possible navel infections. Total serum protein (TSP) was measured using a digital refractometer (MISCO Palm Abbe model PA202X, Solon, OH, USA). The overall mean TSP concentration at enrollment was 5.2 ± 0.63 g/dL (range: 4.0–7.1 g/dL), broadly consistent with adequate passive transfer of immunity (TSP ≥ 5.2 g/dL). A significant interaction between milk replacer and calf starter DFM supplementation was observed for TSP (*p* < 0.05), with calves in the CLP group exhibiting the greatest TSP values. Pre-existing variation in TSP is acknowledged as a potential source of metabolic variability across treatment groups. The overall mean TSP concentration at enrollment was broadly consistent with adequate passive transfer of immunity (TSP ≥ 5.2 g/dL) [16].

Calves were blocked by body weight and TSP, and randomly assigned to 1 of 4 treatments using a randomized complete block design with a 2 × 2 factorial arrangement, with the main factors being separate DFMs in milk replacer (MR) and calf starter (CS). Treatments were assigned as follows: CON (control); MR and CS without DFMs; CLP: MR without DFMs and CS containing 0.02% DFMs (LactoPlan, Nutraferma, Inc., Sioux City, IA, USA); *Bifidobacterium animalis* + *Lactobacillus animalis* in MR (BBCM): MR containing 2 g/calf per day of experimental DFMs and CS without DFMs; and a combination of CLP and BBCM (CMLP): MR and CS, each containing DFMs at specified rates. The milk replacer DFM product was a water-soluble dry powder with a certificate of analysis guaranteeing 2.5 × 10^8^ cfu/g of *Lactobacillus animalis* and 5 × 10^7^ cfu/g *Bifidobacterium animalis* on a dextrose carrier (as-fed basis). The DFMs in CS contained 1.2 × 10^8^ cfu/g heat-stable *Lactobacillus plantarum,* supplying approximately 529,104 CFU per kg CS at a 0.2% inclusion rate. The MR was a 22% CP and 20% fat formulation and contained decoquinate (Deccox, Zoetis Inc., Parsippany, NJ, USA) added at a rate of 41.7 mg/kg (as-is basis) for coccidiosis control, and 16 mg/kg diflubenzuron (ClariFly, Central Life Sciences, Schaumburg, IL, USA) for fly control.

Milk replacer (MR) was fed at 0.56 kg/calf per day from day 1 to 14, then increased to 0.83 kg/calf per day from day 15 to 42 in two equal feedings. From day 43 to 49, MR was reduced to 0.42 kg/calf once daily at morning feeding, after which the calves were weaned. For BBCM and CMLP, the DFM product was mixed with water and delivered daily through day 56 (post-weaning period). All MR was fed at 15% solids, with refusals recorded. From day 1, calves received *ad libitum* water and approximately 24% CP pelleted calf starter (∼4-mm, 23.7 to 24.1% CP), offered throughout the study. The MR was manufactured and supplied by Actus Nutrition (Eden Prairie, MN, USA), and CS was formulated by Casper Ranch (Freeport, IL, USA), manufactured by a commercial feed mill (Ralco Inc., Marshall, MN, USA). All MR and CS were manufactured in sufficient quantities at one time to complete the experiment using the same lots of ingredients.

On day 56, before the morning feeding, blood samples were collected via jugular venipuncture into 10 mL Vacutainer tubes containing sodium heparin (Vacutainer, Becton Dickinson, Franklin Lakes, NJ, USA) and immediately placed on ice. Plasma samples (three per treatment) were obtained within 15 min of collection by centrifugation at 2500× *g* for 20 min at 4 °C and thereafter stored at −80 °C until untargeted metabolomics analysis.

### 2.3. Metabolome Analysis of Blood Plasma

#### 2.3.1. CIL-LC/MS-Based Metabolomics

Untargeted metabolome profiling was conducted using chemical isotope labeling (CIL) coupled with liquid chromatography–mass spectrometry (LC–MS). The CIL approach employed ^12^C/^13^C-dansylation labeling, which enables group-specific detection of metabolites such as amines/phenols, carboxylic acids, carbonyls (aldehydes and ketones), and hydroxyls. Detailed procedures for sample preparation, isotope labeling, and analytical workflow have been previously described by Idowu et al. [17]. In total, 15 raw LC–MS data files were generated, comprising 12 plasma samples (3 replicates per treatment) and 3 pooled quality control samples.

#### 2.3.2. Metabolite Data Processing and Identification

The MS spectral peaks from all samples generated from the LC–MS were first exported with Agilent MassHunter software (v12.0, Agilent Technologies, Santa Clara, CA, USA). The exported data were uploaded to IsoMS Pro 1.2.16 for data quality checks and processing (peak picking, peak pairing, and peak-pair filtering) to remove redundant peaks of the same metabolite, such as adduct ions, dimers, and multimers. Peak pairs that were not present in at least 80% of samples within any group were removed. After filtering, all data were normalized by the ratio of total useful signals. Metabolite identification of the peak pairs was carried out based on mass and retention time matching using the CIL library (https://www.MyCompoundID.org (accessed on 4 April 2024) and based on accurate mass and predicted retention time matches using the linked identity library, which includes over 9000 pathway-related metabolites [18]. All metabolite identifications were reported at Metabolomics Standards Initiative (MSI) Level 2 (putative annotation based on accurate mass and predicted retention time matching), as tandem mass spectrometry with authentic reference standards was not employed. Consequently, isomeric resolution for structurally similar keto-acid metabolites requires future confirmation by MSI Level 1 identification methods.

### 2.4. Statistical and Data Analysis

The final metabolite-intensity data file was exported to Metaboanalyst 6.0 software (https://www.metaboanalyst.ca/ (accessed on 24 July 2024) for statistical analysis. Data were checked for integrity prior to modelling and then data normalization was carried out using median-centering, log transformation and autoscaling. Differentially abundant metabolites among calf groups were determined using *p*-values ≤ 0.05 and area under the curve (AUC) > 0.80 based on the receiver operating characteristic (ROC) curve as calculated by the ROCCET web server after the data were log-transformed, auto-scaled and normalized using median-scale normalization. No significant feature was detected using set parameters when the false discovery rate (FDR) was set at 0.05 and with a fold change of 1.0. Accordingly, the results are presented as exploratory findings based on nominal *p*-values (≤0.05) combined with a fold change threshold (≥1.2 or ≤0.83) and AUC > 0.80, consistent with approaches used in small-sample untargeted metabolomics studies. Independent validation in larger cohorts is required before definitive conclusions can be drawn. To elucidate alterations in metabolic pathways in plasma, a pathway enrichment analysis of the metabolome data was conducted. This analysis aimed to identify metabolic pathways that exhibited significant differences (*p* ≤ 0.05) in calves fed with DFM compared with the CON. For each ROC analysis, sensitivity (true positive rate), specificity (1 minus false positive rate), and Youden’s index (sensitivity + specificity −1) were determined at the optimal classification threshold. It should be noted that AUC values of 1.0 derived from groups of *n* = 3 per group are likely to reflect overfitting; these metabolites are therefore considered exploratory candidate biomarkers requiring independent validation.

## 3. Results

### 3.1. Partial Least Squares–Discriminant Analysis (PLS-DA) Score Plot

**Figure 1:**
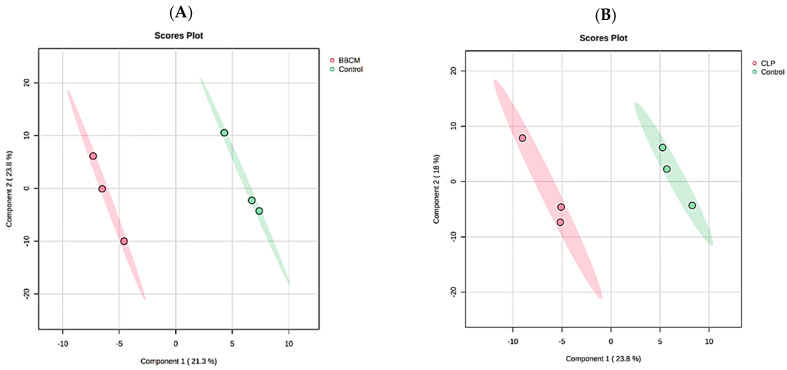

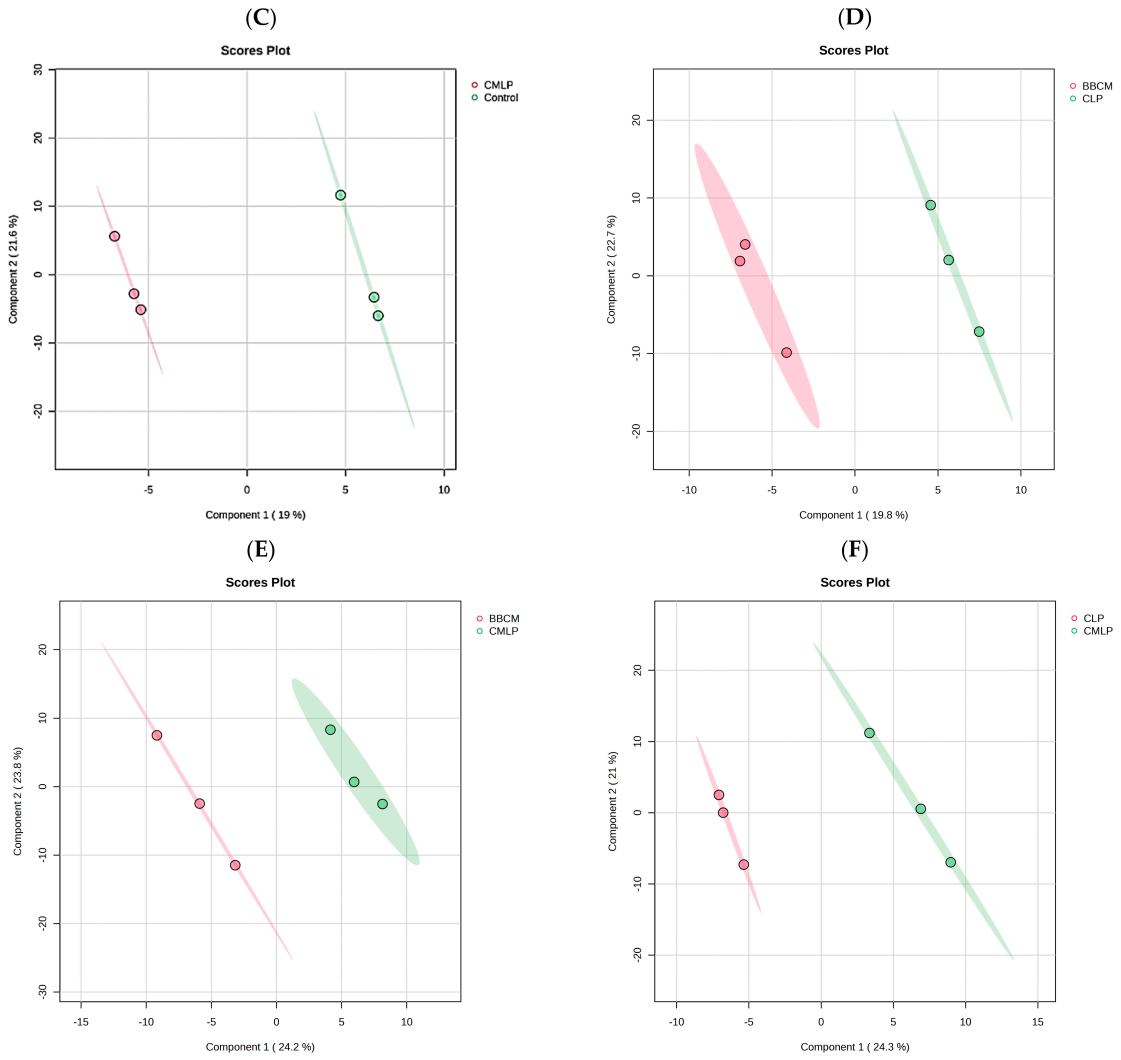
PLS-DA scores plot showing the metabolome between (**A**) BBCM and CON groups; (**B**) CLP and CON groups; (**C**) CMLP and CON groups; (**D**) BBCM and CLP groups; (**E**) BBCM and CMLP groups; (**F**) CLP and CMLP groups.

A total of 231 carboxylic-acid-containing metabolites were detected and identified in the plasma samples of calves. The partial least squares–discriminant analysis (PLS-DA) score plot showed distinct separation between the plasma carboxyl metabolome of the control and treatment groups. The PLS-DA score plots demonstrated clear separation between the control and treatment groups based on the first two principal components, with explained variances of 23.8% and 21.3% for BBCM and CON (Figure 1A), 18.0 and 23.8% for CLP and CON (Figure 1B), 22.2 and 18.4% for CMLP and CON (Figure 1C), 22.7% and 19.8% for BBCM and CLP (Figure 1D), 23.8 and 24.2% for BBCM and CMLP (Figure 1E), and 21 and 24.3% for CLP and CMLP (Figure 1F). These values suggest that the DFM products modified the calf plasma metabolome.

### 3.2. Volcano Plot Analysis of the Differential Plasma Metabolites Between Treatment and Control Groups

**Figure 2:**
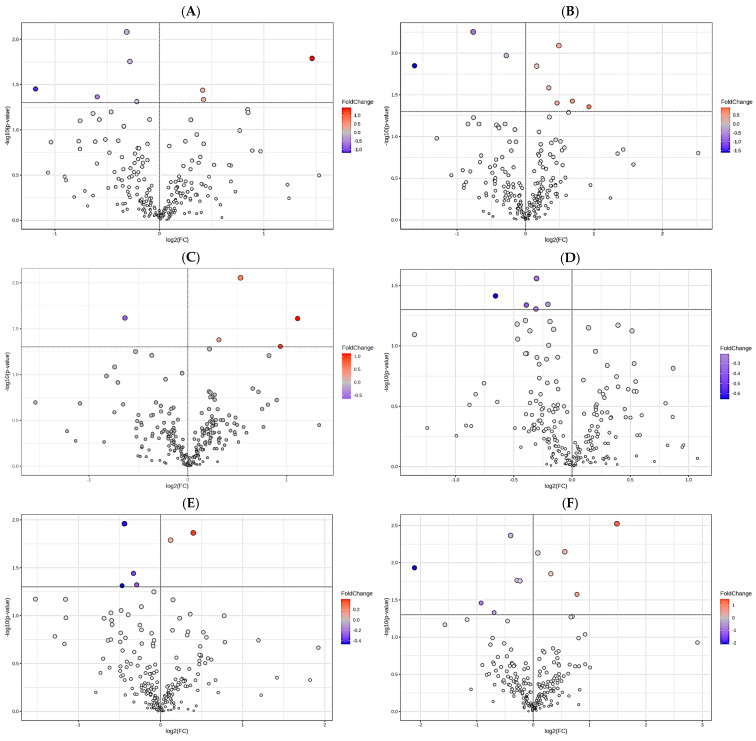
Volcano plot showing differentially abundant metabolites of calves between (**A**) BBCM and CON groups; (**B**) CLP and CON groups; (**C**) CMLP and CON groups; (**D**) BBCM and CLP groups; (**E**) BBCM and CMLP groups; (**F**) CLP and CMLP groups.

Results of the volcano plot analysis (Figure 2A–F) revealed differentially abundant metabolites (FC ≥ 1.2 or ≤ 0.83 and *p*-value ≤ 0.05), with the values of relative abundance of metabolites in the control and treatment groups shown in Table 1, Table 2, Table 3, Table 4, Table 5 and Table 6. The volcano plot in Figure 2A showed that a total of nine differentially abundant metabolites were detected between BBCM and CON. Compared to the CON, three metabolites were significantly (*p* < 0.05) upregulated, while six were downregulated in the CON group. The comparison between the CLP and CON groups showed that nine metabolites were detected as differentially abundant, six were differentially (*p* < 0.05) upregulated, and three were downregulated (Figure 2B). Figure 2C shows that six metabolites were differentially abundant between the CMLP and CON groups, while four were upregulated and two were downregulated. Comparing the BBCM and CLP groups showed that no metabolites were upregulated, while five were downregulated (Figure 2D). Of the seven metabolites that were differentially abundant between BBCM and CMLP (Figure 2E), two were upregulated, while five were downregulated. Twelve (12) were detected as differentially abundant between CLP and CMLP, with five being upregulated and seven being downregulated (Figure 2F).

As shown in Table 1, the fold changes of metabolites in calves from the BBCM group compared with those in the CON group revealed several increases, including in 2D-5-O-methyl-2,3,5/4,6-pentahydroxycyclohexanone (2.75), 5-methyl-5-hexen-2-one (1.34) and rotundifolone (1.33). In contrast, 3-dehydro-L-gulonic acid showed a 0.44-fold decrease, 2-methyl-4-heptanone a 0.80-fold decrease, and molybdopterin precursor Z a 0.66-fold decrease.

As shown in Table 2, calves in the CLP group showed increased abundance of 3-methyl-2-oxo-valeric acid, with a 1.40-fold increase; aldehydo-D-mannose, with a 1.61-fold increase; and keto-D-fructose, with a 1.90-fold increase. On the other hand, molybdopterin precursor Z showed a 0.59-fold decrease, and 2-hydroxy-3-methylbenzalpyruvic acid showed a 0.33-fold decrease.

In the CMLP group (Table 3), rotundifolone exhibited a 1.45-fold increase, 2D-5-O-methyl-2,3,5/4,6-pentahydroxycyclohexanone a 2.16-fold increase, 3-methyl-2-oxo-valeric acid a 1.24-fold increase, and furostanol-26-aldehyde a 1.91-fold increase. In contrast, (11R)-dihydroartemisinic aldehyde and 2-trans,6-trans-farnesal both had a 0.64-fold decrease.

All differential metabolites detected between the BBCM and CLP groups had a fold change decrease (Table 4). (-)-Fenchone had a 0.81-fold decrease, xanthoxin a 0.63-fold decrease, (2E,4E,6E)-7-hydroxy-4-methylhepta-2,4,6-trienal a 0.76-fold decrease, and (2E,6Z)-non-2,6-dienal a 0.81-fold decrease. The comparison between the BBCM and CMLP groups showed that (2E,4E,6E)-4-methylocta-2,4,6-trienedial had a 1.32-fold increase, while decreases were observed in isomer 1 of 2-hydroxy-6-oxo-6-(4′-chlorophenyl)-hexa-2,4-dienoic (0.74-fold) and coniferyl aldehyde (0.72-fold) (Table 5).

Comparison of differential metabolites between the CLP and CMLP groups (Table 6) showed that 6-demethylgriseofulvin had a 2.80-fold increase, ribulose a 1.48-fold increase, and 2-methyl-3-oxopropanoic acid a 1.71-fold increase in the CLP group. In contrast, 3alpha,7alpha,12alpha-trihydroxy-5beta-cholestan-26-al had a 0.76-fold decrease, 2-hydroxy-3-methylbenzalpyruvic acid a 0.23-fold decrease, 16alpha-ghdroxyandrost-4-ene-3,17-dione 16-O-glucuronide a 0.53-fold decrease, and 1,2-benzoquinone a 0.62-fold decrease.

### 3.3. Biomarkers with AUC Values

**Figure 3:**
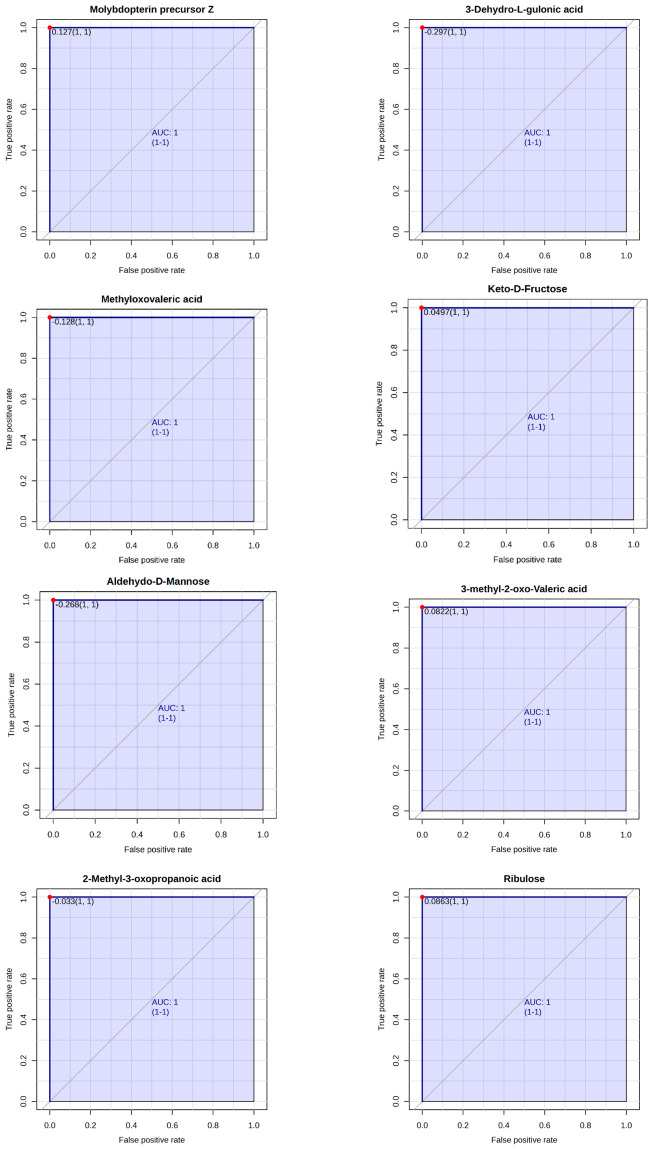
Receiver operating characteristic curves of biomarkers enhanced by the treatment groups.

The plasma metabolites that served as biomarkers were assessed using receiver operating characteristic (ROC) curves, with sample data shown in Figure 3. Based on the established biomarker utility classification, candidate markers with an AUC of 0.9 or higher are considered “excellent.” In this study, some plasma metabolites that are candidate biomarkers (AUC = 1.0) are listed in Table 7. It should be noted that AUC values of 1.0 obtained from comparisons of groups with *n* = 3 per group are likely to reflect overfitting due to the limited sample size rather than true perfect discriminatory capacity. These metabolites are therefore considered exploratory candidate biomarkers requiring independent validation in adequately powered cohorts.

### 3.4. Metabolite Set Enrichment Analysis of Differentially Abundant Metabolites

**Figure 4:**
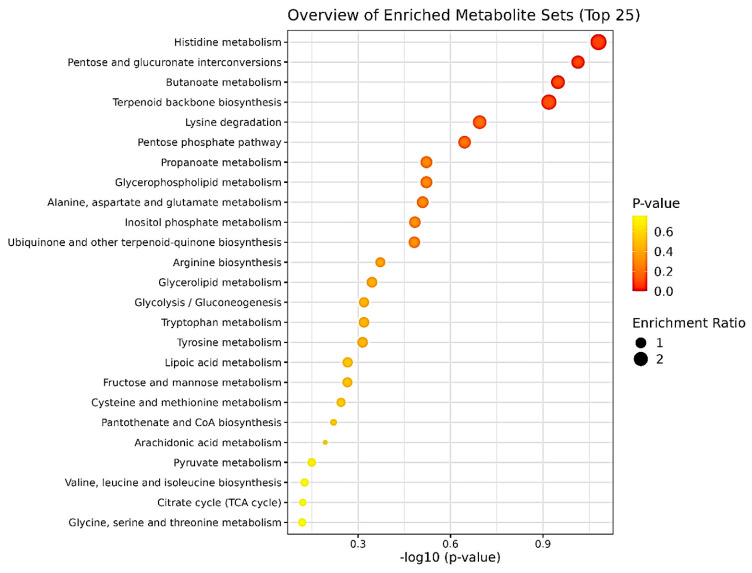
Metabolite set enrichment analysis of calves in the BBCM and CON groups.

**Figure 5:**
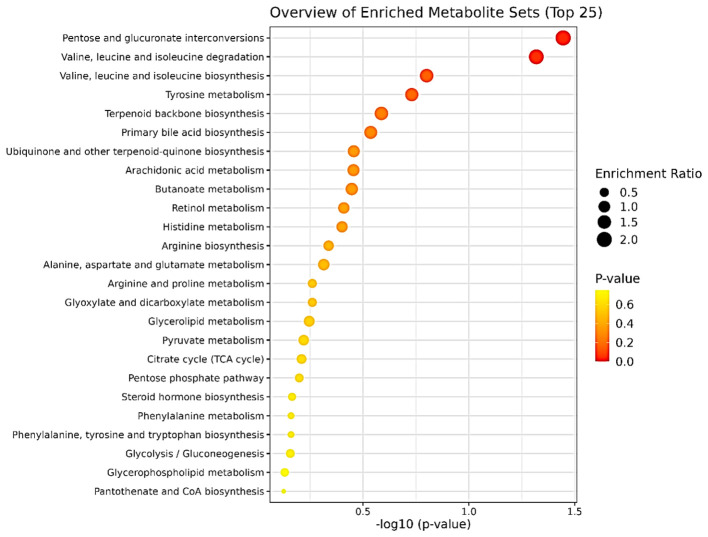
Metabolite set enrichment analysis of calves in the CLP and CON groups.

**Figure 6:**
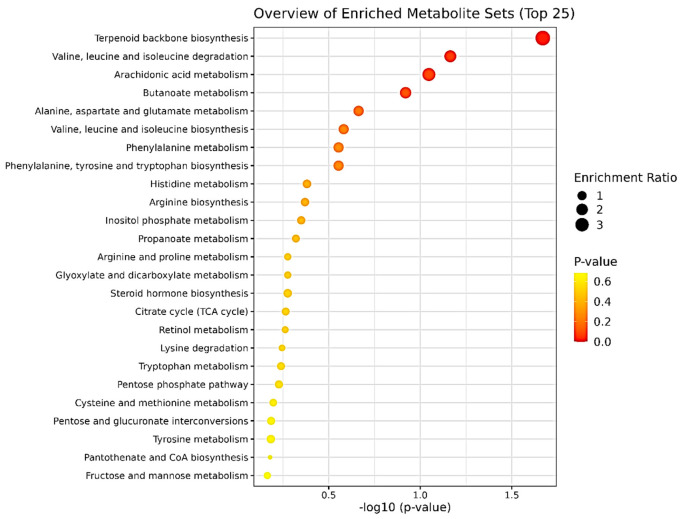
Metabolite set enrichment analysis of calves in the CMLP and CON groups.

**Figure 7:**
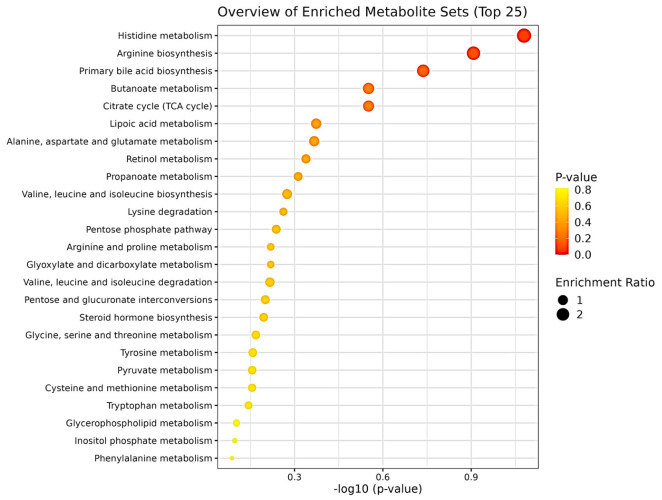
Metabolite set enrichment analysis of calves in the BBCM and CLP groups.

**Figure 8:**
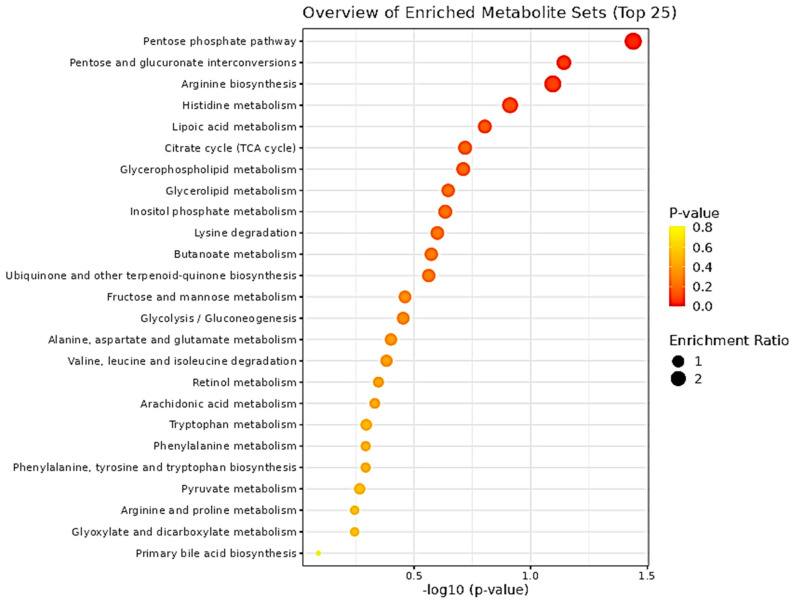
Metabolite set enrichment analysis of calves in the BBCM and CMLP groups.

**Figure 9:**
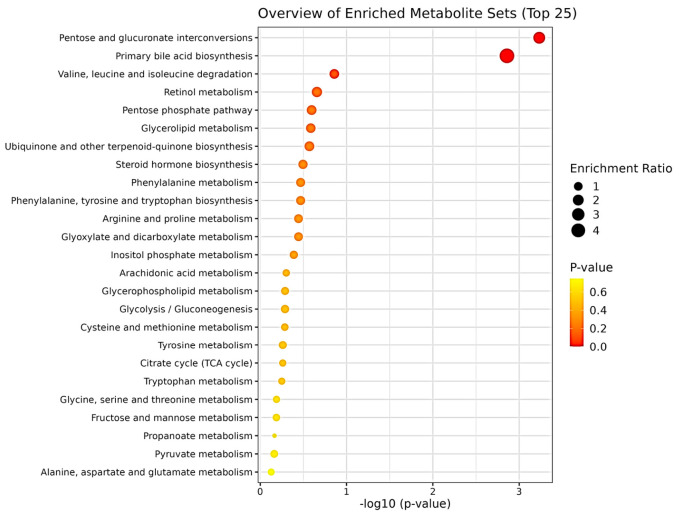
Metabolite set enrichment analysis of calves in the CLP and CMLP groups.

Results of the metabolite set enrichment analysis, identifying key metabolic pathways that were significantly altered between calves in the treatment groups and the control group, are presented in this section. The enrichment pathway analysis revealed that BBCM treatment led to the enrichment of histidine metabolism, pentose and glucuronate interconversions, butanoate, and terpenoid backbone biosynthesis metabolic pathways (Figure 4). Calves in the CLP group showed enrichment of key metabolic pathways, including pentose and glucuronate interconversions, and valine, leucine and isoleucine degradation (Figure 5). Pathways enriched in calves in the CMLP group include terpenoid backbone biosynthesis; valine, leucine and isoleucine degradation; and arachidonic acid metabolism (Figure 6). Comparison between BBCM and CLP showed the enrichment of pathways, including histidine metabolism, arginine biosynthesis and primary bile acid biosynthesis (Figure 7). In Figure 8, the analysis showed that the comparison between BBCM and CMLP had a greater enrichment ratio of pentose phosphate pathway, pentose and glucuronate interconversions, arginine biosynthesis and histidine metabolism. Pentose and glucuronate interconversions and primary bile acid biosynthesis were the pathways that showed the greatest enrichment when comparing the CLP and CMLP groups (Figure 9).

## 4. Discussion

Significant physiological and metabolic changes characterize the development of pre-weaned calves, especially as they transition from milk to a solid diet, a period that transforms their digestion model from nominally monogastric organisms to functional ruminants. These changes are coupled with health challenges like diarrhea and respiratory difficulties due to the immaturity of the digestive system and immune system, making them much more susceptible to the external environment [19,20]. Neonatal calf diarrhea is usually followed by a negative energy balance characterized biochemically by a reduction in glucose concentration [21]. In another study where anorexia was observed in diarrheic calves, decreased levels of glucose showed a negative correlation with DL-lactate, which is thought to result in poor nutrient absorption [11].

The direct-fed microbial products that were fed individually in milk replacer or calf starter, or combined, influenced the plasma metabolite profiles, which indicated specific alterations in the physiological pathways linked to energy and amino acid metabolism in calves. The group of calves fed milk replacers containing BBCM had increased abundance of 5-methyl-5-hexen-2-one, a molecule that may be associated with metabolic and energy-related biological processes. Ketone-containing small molecules such as 5-methyl-5-hexen-2-one may participate in intracellular signaling cascades, though direct evidence for this compound’s role in bovine physiology is currently limited. There was a downregulation of 3-dehydro-L-gulonic acid (0.44 fold), which is often identified in metabolic pathways as 2,3-diketo-L-gulonate or DKG. The compound is a signaling molecule for cellular stress, being a major irreversible degradation product of vitamin C in blood plasma, and it acts as an intermediate in the breakdown of ascorbate. It potentially generates hydrogen peroxide, acting as an antioxidant by protecting lipoproteins [22].

Upregulation of carbohydrate and protein metabolism-related compounds was observed in the plasma of calves fed plain milk replacer with a starter diet containing CLP. Keto-D-fructose, which increased by 1.90-fold, could indicate better carbohydrate utilization in calves. It is typically referred to as fructose or fructose-1-phosphate and is highly relevant to glycolytic catabolism entering the pathway, bypassing the rate-limiting phosphofructokinase-1 step that regulates glucose metabolism. The continuous conversion of fructose to triose phosphates (DHAP and glyceraldehyde) allows for rapid, unregulated, and sustained conversion into energy and fat [23]. The upregulation of this compound, which ensures continuous high levels of cellular ATP, might be important for the survival and adaptation of pre-weaned calves when they experience negative energy balance during diarrheic conditions while feeding on milk. Another importance of keto-D-fructose lies in the synthesis and degradation of D-sorbitol by gut bacteria, a process that regulates energy metabolism and protects against fatty liver disease, forming a critical protective link in the gut–liver axis [24]. *Escherichia coli* (Enterobacteriaceae) metabolizes sorbitol in the gut as a carbon source, reducing the osmotic pressure in the colon and suppressing sorbitol-induced diarrhea [25].

The upregulation of another carbohydrate compound, aldehydo-D-mannose (1.61 fold), could signal a health benefit for calves, and this is supported by emerging evidence that it demonstrates anti-diabetic and anti-inflammation bioactivities. The anti-inflammatory effect is dependent on its transport into mammalian cells, where it promotes the differentiation of regulatory T cells (Tregs), and regulates the activation of hepatic stellate cells (HSCs) and macrophages, partially via modulation of glucose utilization [26]. According to Hu et al. [27], D-mannose can stimulate insulin secretion through the capacity of its α-anomer to provoke the accumulation of aldohexose-bisphosphates in pancreatic islets. Additionally, D-mannose is an important metabolic intermediate in N-glycosylation in the biosynthesis of glycoproteins, in which secretory proteins are covalently attached to carbohydrate chains. These proteins are essential for protein stability, cell communication, and signaling, highlighting the beneficial effects of d-mannose on the immune system, and against metabolic syndrome and intestinal diseases [28].

The detection and increased concentration of 3-methyl-2-oxo-valeric acid (1.40-fold), shows increased activity in isoleucine metabolism. 3-Methyl-2-oxovaleric acid is produced from isoleucine by cytosolic branched-chain aminotransferase 1 enzyme, and it is a 2-oxo monocarboxylic acid, which is a derivative valeric acid carrying oxo- and methyl substituents at C-2 and C-3, respectively. It is used as a clinical marker for maple syrup urine disease (MSUD). It is functionally related to valeric acid. The isomer of the identified compound is 2-methyl-3-ketovaleric acid, a metabolite of leucine in the branched-chain keto-acid pathway of leucine metabolism, and it is a known pathological metabolite and is associated with propionic acidemia, especially during periods of ketoacidosis detected in some neonatal cases. 2-Methyl-3-ketovaleric acid induces a strong activity of inosine-5′-monophosphate dehydrogenase, the rate-limiting enzyme in guanosine-5′-triphosphate synthesis, mimicking glucose [29].

Gut bacterial activity is marked by the presence of (R/S)-acetoin, with an observed upregulation of 1.12-fold. Also known as 3-hydroxy-2-butanone, the four-carbon metabolic intermediate compound produced in various bacteria during fermentative metabolism acts as a neutral metabolic intermediate, with its production proposed to moderate environmental acidification by diverting carbon flux away from organic acids. Because intestinal pH and microbiome data were not collected in the present study, the mechanistic relationship between plasma acetoin concentration and intestinal acid regulation remains to be determined [30]. It is produced via the condensation of pyruvate, which helps manage NADH/NAD+ balances and diverts carbon flux away from acidic byproducts [31].

Calf plasma of those in the CMLP group revealed detectable increased levels of D-5-O-methyl-2,3,5/4,6-pentahydroxycyclohexanone by 2.16-fold. The keto intermediate in pinitol biosynthesis II [32], a pathway observed in bacteria, fungi, algae, and plants that functions in osmoregulation and osmo-protection, is formed from sequoyitol through the action of NAD- and NADP-dependent dehydrogenases [32]. It should be noted that rotundifolone, furostanol-26-aldehyde, and xanthoxin are plant-derived secondary metabolites whose presence in calf plasma most likely reflects absorption of phytochemical constituents from calf starter ingredients rather than endogenous host metabolic activity. Their differential abundance should therefore be interpreted as a putative dietary signal rather than evidence of direct DFM-mediated modulation of host physiology.

The reappearance of 3-methyl-2-oxo-valeric acid in the plasma of calves in the CMLP group (1.24 fold), similar to the CLP, where *Lactobacillus plantarum* is present in the starter feed, demonstrates a consistent treatment effect that promotes metabolites involved in the isoleucine and leucine metabolic pathways, respectively. These compounds reflect branched-chain amino acid catabolism and are associated with mitochondrial function, insulin resistance, and the regulation of brown adipose tissue activity.

Metabolite set enrichment analysis transforms the identified metabolite concentrations into biologically meaningful insights by interpreting and prioritizing statistically significant metabolic pathways, functional groups, or disease-related patterns, which allows for the understanding of underlying biological mechanisms rather than just identifying individual biomarkers. Enriched pathway analysis that was unique to the treatments included histidine metabolism, lysine degradation, pentose phosphate pathway (e.g., BBCM), valine, leucine and isoleucine biosynthesis, tyrosine metabolism (e.g., CLP), and arachidonic acid metabolism (e.g., CMLP). Pentose and glucuronate interconversions were common in BBCM and CLP, butanoate metabolism and terpenoid backbone biosynthesis were common in BBCM and CMLP, and valine, leucine and isoleucine degradation pathway was common in CLP and CMLP.

Pentose and glucuronate interconversions are an important carbohydrate metabolic pathway involved in interconversions of pentose and glucuronate, the salts or esters of glucuronic acid [33]. This pathway plays a key role in biosynthetic processes, including gluconeogenesis, transamination, deamination, and lipogenesis; aberrant activity is associated with diseases such as familial tumoral calcinosis [34]. It is the most relevant pathway in disease progression, where D-glucuronic acid binds toxic substances via UDP–glucuronosyltransferase to increase water solubility for excretion in bile or urine [35]. High-throughput analysis of pathway metabolites enables biomarker discovery, disease diagnostics, drug discovery, food safety, and metabolic disease research.

Histidine can be first-limiting for growth; in cattle fed maize silage, the ruminal and dietary supply was insufficient to maintain growth in young bulls [36]. Histidine is a dietary-essential amino acid that is not synthesized de novo and must be obtained through the diet [36,37]. It is one of the least abundant amino acids in whole-body protein in humans [37]. Its deficiency decreases body weight, amino acid oxidation, and protein turnover, making it indispensable for healthy adults and ruminants [36]. Functionally, histidine supports growth, tissue repair, blood cell production, and nerve protection [38]. It forms metal ion complexes, such as the axial base to Fe in myoglobin and hemoglobin [38]. Histidine is enzymatically decarboxylated to histamine, a neurotransmitter involved in immune response, gastric acid secretion, and brain function, and mediating immune cell growth and functionality [37,38,39]. It also reverses high-salt-induced hepatic metabolic disorders, ameliorates liver amino acid metabolism, enhances enzymatic and glutathione antioxidant systems, and promotes nitric oxide production, which protects cell integrity during oxidative stress [40].

Butanoate metabolism, or butyrate metabolism, in neonatal calves primarily reflects intestinal (hindgut) fermentation of carbohydrates by gut microbiota to produce butyric acid, a four-carbon short-chain fatty acid, particularly during the developmental stage, when the rumen is not yet fully functional [41]. As a main product of this microbial fermentation, butyrate mediates the gut microbiota regulation of whole-body energy homeostasis [42]. It exerts anti-inflammatory effects by increasing SCFA-producing bacteria and decreasing endotoxin-secreting bacteria [43], and alterations in butyrate-producing bacteria are linked to ulcerative colitis and type II diabetes [44]. Butanoate metabolites increase energy expenditure and reduce adiposity [45]. In calves, butyrate promotes gastrointestinal and rumen development; improves digestion of fat, lactose, and protein, enhances feed efficiency; reduces diarrhea; improves growth; and provides antimicrobial protection [46,47]. It inhibits inflammation, enhances immunity and energy harvesting, and activates microbial carbohydrate metabolism [46]. Butyrate regulates metabolism, aids transepithelial fluid transport, induces epithelial defense, and ameliorates insulin resistance, obesity, and liver injury [48]. It also decreases oxidative damage to colorectal cells and alleviates oxidative stress; sodium butyrate supplementation boosts the oxidative stress defense system by increasing GSH-Px activity and lowering serum MDA [49].

Terpenoids are bioactive compounds in essential oils, with terpenes and terpenoids exhibiting anticancer, antimicrobial, anti-inflammatory, antioxidant, and antiallergic activities [50]. Produced by aromatic and medicinal plants, they aid in disease resistance; monoterpenoids disrupt microbial multiplication and physiological activity [50]. In pigs exposed to maternal immune activation and weaned, the terpenoid backbone biosynthesis pathway was enriched, and its gene expression patterns have advanced understanding of therapeutic effects on hippocampus-related disorders [51]. According to the Kyoto Encyclopedia of Genes and Genomes (KEGG), the terpenoid backbone biosynthesis pathway encompasses the formation of isoprenoid precursors, including isopentenyl diphosphate (IPP) and dimethylallyl diphosphate (DMAPP), which are further condensed into geranyl, farnesyl and geranylgeranyl diphosphates [52]. These intermediates constitute the core substrates for the biosynthesis of sterols, carotenoids, ubiquinone and dolichol, which are essential for membrane integrity, antioxidant defense and mitochondrial electron transport. In the present study, enrichment of this pathway in the BBCM group may reflect microbiota-associated modulation of host isoprenoid metabolism, potentially influencing cellular redox balance and energy metabolism in pre-weaned calves. However, given the untargeted nature of the metabolomics approach, these interpretations remain putative and hypothesis-generating rather than mechanistically confirmed.

The enrichment of the pathway for valine, leucine and isoleucine biosynthesis was observed in CLP calves, and these branched-chain amino acids (BCAAs) are significant for improving stress resistance, maximizing the production of essential amino acids in industrial microorganisms, and managing metabolic health in animals. BCAAs are essential to activating the mechanistic Target of Rapamycin (mTOR) signaling pathway, which connects diverse physiological roles, such as protein synthesis, autophagy, and glucose homeostasis [53]. On the other hand, the degradation pathways of the BCAAs were also enriched in CLP and CMLP. Leucine is particularly important for mTOR in its role as a central kinase for regulating neurotransmission, immune response, intestinal development, mitochondrial biogenesis, and milk production [54,55]. BCAA degradation is also critical for energy production and nitrogen metabolism, particularly in skeletal muscle, converting valine, leucine, and isoleucine into acetyl-CoA, succinyl-CoA, and acetoacetate to fuel the tricarboxylic acid cycle. An enriched BCAA degradation pathway indicates active catabolism for energy during metabolic stress, fasting, or high muscle protein turnover. This process generates NADH, FADH2, and ATP precursors that support immune function and skeletal muscle development during critical early life [56]. It should be noted that the identification of the keto-acid intermediates supporting this pathway enrichment is at MSI Level 2, and isomeric resolution between 3-methyl-2-oxo-valeric acid and structurally related species requires confirmation by tandem mass spectrometry with authentic reference standards.

Tyrosine metabolism was enriched in CLP calves, identifying a pathway that is marked by changes in pre-weaned calves during weaning, driven by diet shifts from milk to solid feed and associated stress [57]. Plasma tyrosine, a key metabolic indicator alongside phenylalanine and tryptophan, decreases in response to abrupt weaning as the rumen develops [57]. In diarrheic calves, tyrosine metabolism is altered, reflecting intestinal dysfunction and microbial shifts, with reduced energy metabolites, amino acids, and changes in biogenic amines [10]. Tyrosine abundance decreased in weaned calves during the first 2 days post-weaning, suggesting its role in stress response. The observations in this study showed that calves that received *Lactobacillus plantarum* had abundant levels compared to the control. As a precursor to catecholamine neurotransmitters dopamine, norepinephrine, and epinephrine, acute weaning stress likely increases tyrosine demand and utilization, reducing blood concentrations [58,59].

Arachidonic acid is metabolized by cyclooxygenases, lipoxygenases, and cytochrome P450 enzymes to generate diverse bioactive lipid mediators [60] and was detected as being enriched in CMLP calves. It is the direct precursor of eicosanoids such as prostaglandins, leukotrienes, and epoxyeicosatrienoic acids, which are involved in cell differentiation, tissue development, and organ function, but also in hepatic fibrosis, neurodegeneration, obesity, diabetes, and cancers [61]. The arachidonic acid metabolic network produces inflammatory mediators that are widely involved in immune and inflammatory responses, linking metabolism with immunity [62]. These eicosanoids are generally proinflammatory, triggering oxidative stress, stimulating immune responses, and promoting inflammation in tissues, including the kidney [4,61]. Thus, enrichment in arachidonic acid pathways is an effective strategy for managing inflammation-related diseases [61].

Several limitations of this study warrant acknowledgment. Although metabolite annotation employed a dual-criterion approach combining accurate mass and retention-time matching, supported by the additional selectivity of chemical isotope labeling, metabolite identifications remain at MSI Level 2 confidence because they were not validated using authentic reference standards. Consequently, the annotated metabolites should be considered putative identifications, and targeted LC-MS/MS analyses with authentic standards are required to confirm key metabolites, particularly the structurally similar BCAA catabolites, and to achieve MSI Level 1 identification confidence. In addition, the metabolomics substudy was conducted using a limited number of samples per treatment group (*n* = 3), which reduced statistical power following multiple-testing correction and may have increased the risk of overfitting in AUC-based biomarker analyses. Furthermore, a subset of the differentially abundant features, including rotundifolone, furostanol-26-aldehyde, and xanthoxin, likely represent dietary phytochemicals or other exogenous compounds rather than endogenous mammalian metabolites; therefore, their interpretation as biomarkers of host metabolic responses should be made with caution. Finally, the absence of complementary measurements such as gastrointestinal pH, microbial community composition and short-chain fatty acid concentrations precludes definitive mechanistic conclusions regarding the effects of direct-fed microbials on gut fermentation and host metabolism. Future studies incorporating larger sample sizes, targeted metabolite validation, and integrated microbiome and fermentation analyses will be necessary to confirm and extend these exploratory findings.

## 5. Conclusions

This study evaluated the effects of direct-fed microbials (DFMs) on the plasma metabolome of pre-weaned dairy bull calves using untargeted chemical isotope labeling liquid chromatography–mass spectrometry (CIL-LC/MS). Direct-fed microbial supplementation modulated the plasma metabolome of dairy bull calves by altering the abundance and activity of pathways involved in amino acid and carbohydrate metabolism. Administration of *Lactobacillus animalis*, *Bifidobacterium animalis*, *Lactobacillus plantarum*, or their combination through milk replacer and/or calf starter feed resulted in distinct plasma metabolite profiles, indicating that these DFM formulations influenced systemic metabolism and nutrient utilization during early life. Significant enrichment was observed in histidine, leucine, valine, isoleucine and tyrosine metabolism, as well as in butyrate, arachidonic acid, and carbohydrate metabolism pathways. Differentially abundant metabolites included increased concentrations of 3-methyl-2-oxo-valeric acid, methyloxovaleric acid, aldehydo-D-mannose and keto-D-fructose, and decreased concentrations of 3-dehydro-L-gulonic acid. Observed changes were associated with metabolic processes related to branched-chain amino acid metabolism, bacterial fermentation, N-glycosylation, carotenoid metabolism and glycolysis. By identifying differentially abundant metabolites and the metabolic pathways enriched in response to DFM supplementation, this study provides evidence that DFM-induced modulation may contribute to enhancing the metabolism of pre-weaned calves. Collectively, the findings support the hypothesis that DFM supplementation modulates plasma metabolic profiles and associated plasma metabolite profiles and associated pathways during early life.

## Figures and Tables

**Table 1:** Differentially abundant plasma metabolites between the BBCM and CON groups.

Metabolites	FC	log2 (FC)	*p* Value	FDR
Nonanal	0.80	−0.31	0.008	0.153
2-Methyl-4-heptanone	0.80	−0.31	0.008	0.153
2D-5-O-Methyl-2,3,5/4,6-Pentahydroxycyclohexanone	2.75	1.46	0.016	0.356
2-Tridecanone	0.82	−0.28	0.017	0.356
3-Dehydro-L-gulonic acid	0.44	−1.19	0.035	0.356
Rotundifolone	1.33	0.41	0.036	0.660
Molybdopterin precursor Z	0.66	−0.60	0.043	0.661
5-Methyl-5-hexen-2-one	1.34	0.42	0.046	0.147
Methylimidazole acetaldehyde	0.86	−0.22	0.048	0.356

**Table 2:** Differentially abundant plasma metabolites between the CLP and CON groups.

Metabolites	FC	log2 (FC)	*p* Value	FDR
Molybdopterin precursor Z	0.59	−0.76	0.006	0.287
3-methyl-2-oxo-Valeric acid	1.40	0.49	0.008	0.287
Heptadecatrienal	0.82	−0.28	0.010	0.665
2-Hydroxy-3-methylbenzalpyruvic acid	0.33	−1.62	0.014	0.287
(R/S)-Acetoin	1.12	0.16	0.014	0.287
Methyloxovaleric acid	1.27	0.34	0.026	0.665
Aldehydo-D-mannose	1.61	0.68	0.038	0.702
Xanthoxin	1.38	0.46	0.040	0.702
Keto-D-fructose	1.90	0.93	0.044	0.702

**Table 3:** Differentially abundant plasma metabolites between the CMLP and CON groups.

Metabolites	FC	log2 (FC)	*p* Value	FDR
Rotundifolone	1.45	0.53	0.009	0.885
(11R)-Dihydroartemisinic aldehyde	0.64	−0.64	0.024	0.885
2-Trans,6-trans-farnesal	0.64	−0.64	0.024	0.885
2D-5-O-Methyl-2,3,5/4,6-pentahydroxycyclohexanone	2.16	1.11	0.025	0.885
3-Methyl-2-oxo-valeric acid	1.24	0.32	0.042	0.885
Furostanol-26-aldehyde	1.91	0.94	0.049	0.885

**Table 4:** Differentially abundant plasma metabolites between the BBCM and CLP groups.

Metabolites	FC	log2 (FC)	*p* Value	FDR
(-)-Fenchone	0.81	−0.30	0.028	0.983
Xanthoxin	0.63	−0.66	0.039	0.983
3,4-Dihydroxybenzaldehyde	0.87	−0.21	0.045	0.983
(2E,4E,6E)-7-Hydroxy-4-methylhepta-2,4,6-Trienal	0.76	−0.39	0.046	0.983
(2E,6Z)-Non-2,6-dienal	0.81	−0.31	0.049	0.983

**Table 5:** Differentially abundant plasma metabolites between the BBCM and CMLP groups.

Metabolites	FC	log2 (FC)	*p* Value	FDR
Isomer 1 of 2-hydroxy-6-oxo-6-(4′-chlorophenyl)-hexa-2,4-dienoic acid	0.74	−0.44	0.011	0.844
(2E,4E,6E)-4-Methylocta-2,4,6-trienedial	1.32	0.40	0.014	0.844
Adipate semialdehyde	1.09	0.12	0.016	0.844
(-)-Fenchone	0.80	−0.33	0.036	0.844
2-Methyl-4-heptanone	0.82	−0.29	0.048	0.844
Nonanal	0.82	−0.29	0.048	0.844
Coniferyl aldehyde	0.72	−0.45	0.049	0.844

**Table 6:** Differentially abundant plasma metabolites between the CLP and CMLP groups.

Metabolites	FC	log2 (FC)	*p* Value	FDR
6-Demethylgriseofulvin	2.80	1.49	0.003	0.038
3alpha,7alpha,12alpha-Trihydroxy-5beta-cholestan-26-al	0.76	−0.40	0.004	0.375
Ribulose	1.48	0.56	0.007	0.375
Butanone	1.06	0.083	0.007	0.538
2-Hydroxy-3-methylbenzalpyruvic acid	0.23	−2.10	0.011	0.375
D-Arabinose	1.24	0.31	0.014	0.466
2-Dehydrolubimin	0.82	−0.29	0.017	0.555
Iso-valeraldehyde	0.85	−0.23	0.018	0.466
1-Pentan-3-one	0.85	−0.23	0.018	0.466
2-Methyl-3-oxopropanoic acid	1.71	0.78	0.027	0.555
16alpha-Hydroxyandrost-4-ene-3,17-dione 16-O-glucuronide	0.53	−0.92	0.034	0.466
1,2-Benzoquinone	0.62	−0.69	0.047	0.703

**Table 7:** Biomarkers with AUC values of 1.0.

Name	AUC	*p*-Value	Log2 FC
BBCM			
Nonanal	1	0.004	−0.35
2-Methyl-4-heptanone	1	0.004	−0.35
2D-5-O-Methyl-2,3,5/4,6-pentahydroxycyclohexanone	1	0.006	1.41
3-Dehydro-L-gulonic acid	1	0.008	−1.22
2-Tridecanone	1	0.014	−0.33
Keratan sulfate II (core 2-linked), degradation product 1	1	0.022	−0.51
Molybdopterin precursor Z	1	0.029	−0.64
Rotundifolone	1	0.033	0.37
3-Hydroxy-5-oxohexanoic acid	1	0.041	−0.68
5-Methyl-5-hexen-2-one	1	0.042	0.38
Methylimidazole acetaldehyde	1	0.049	−0.26
Furostanol-26-aldehyde	1	0.049	0.80
CLP			
Methyloxovaleric acid	1	0.009	0.07
(R/S)-Acetoin	1	0.044	−0.49
3-Methoxy-4-hydroxyphenylglycolaldehyde	1	0.031	−0.53
2-Oxovaleric acid	1	0.045	−0.48
Decanal/2-decanone	1	0.038	0.36
Xanthoxin	1	0.004	−1.71
2-Hydroxy-3-methylbenzalpyruvic acid	1	0.010	−0.38
Heptadecatrienal	1	0.033	0.54
2-Deoxy-scyllo-inosose	1	0.007	0.39
3-Methyl-2-oxo-valeric acid	1	0.004	−0.86
Molybdopterin precursor Z	1	0.043	−0.40
(S)-Pyrethrolone	1	0.037	0.83
Keto-D-fructose	1	0.028	0.59
Aldehydo-D-mannose	1	0.021	−0.86
11beta,21-Dihydroxy-5beta-pregnane-3,20-dione 21-O-sulfate	1	0.020	0.24
CMLP			
(11R)-Dihydroartemisinic aldehyde	1	0.017	−0.66
2D-5-O-Methyl-2,3,5/4,6-pentahydroxycyclohexanone	1	0.015	1.09
3-Methyl-2-oxo-valeric acid	1	0.049	0.31
2-Methyl-3-oxopropanoic acid	1	0.025	−0.53
2-Trans,6-trans-farnesal	1	0.017	−0.66
5-Dehydro-4-deoxy-D-glucuronic acid	1	0.029	−0.38
Rotundifolone	1	0.008	0.53

## Data Availability

The original contributions presented in this study are included in the article/Supplementary Material. Further inquiries can be directed to the corresponding author.

## Supplementary Materials

The following supporting information can be downloaded at: https://www.mdpi.com/article/10.3390/metabo16070441/s1, Table S1: Raw metabolome data.

## Abbreviations

The following abbreviations are used in this manuscript:

AUC	Area Under the Curve
BBCM	*Bifidobacterium animalis* + *Lactobacillus animalis* in Milk Replacer
BCAAs	Branched-Chain Amino Acids
CIL	Chemical Isotope Labeling
CLP	*Lactobacillus plantarum*
CMLP	Combination of CLP and BBCM
CON	Control Treatment
CS	Calf Starter
DFMs	Direct-Fed Microbials
FC	Fold Change
KEGG	Kyoto Encyclopedia of Genes and Genomes
LC–MS	Liquid Chromatography–Mass Spectrometry
MR	Milk Replacer
MS	Mass Spectrometry
MSI	Metabolomics Standards Initiative
PLS-DA	Partial Least Squares–Discriminant Analysis
ROC	Receiver Operating Characteristic
TSP	Total Serum Protein
